# WHO Western Pacific Regional Action Plan for the Prevention and Control of NCDs (2014-2020)

**DOI:** 10.4178/epih/e2014007

**Published:** 2014-07-22

**Authors:** Hai-Rim Shin, Cherian Varghese

**Affiliations:** Noncommunicable Diseases and Health Promotion Unit, World Health Organization, Western Pacific Regional Office, Manila, Philippines

This is an excerpt of the “Western Pacific Regional Action Plan for the Prevention and Control of Noncommunicable Diseases (2014-2020)” (RAP NCD) which has been endorsed by the Member States of the Western Pacific Region of World Health Organization (WHO) in October 2013. The Regional plan is fully harmonized with the Global Action Plan for the Prevention and Control of Noncommunicable Diseases (2013-2020). The regional plan calls for a systematic approach to NCD prevention and control. The plan provides a road map and a menu of very cost-effective interventions for all Member States and other stakeholders, to take coordinated and coherent action at all levels to attain the nine voluntary global target by 2025. The original version of RAP NCD is downloadable from the website (http://www.wpro.who.int/noncommunicable_diseases/WHO_NCD_RAP.pdf?ua=1).

In addition, there are NCD tools in the WHO homepage (http://www.who.int/nmh/ncd-tools/en/).

**Figure:**
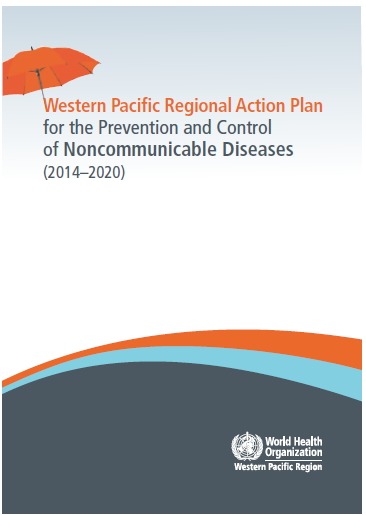


## INTRODUCTION

The noncommunicable disease (NCD) epidemic is a serious threat to life, health and development in the Western Pacific Region. The major NCDs — cardiovascular diseases, diabetes, cancers and chronic respiratory diseases — account for more than 80% of all deaths in the Region. NCDs account for 50% of all premature mortality (under 70 years of age) in low- and middle-income countries in the Region.

In the Region, the burden of morbidity and mortality from NCDs occurs against a complex backdrop of globalization, rapid economic growth, unplanned urbanization, environmental degradation, climate change and growing inequities within countries. Changing lifestyles and increased purchasing power in some populations have led to a reduction in breastfeeding, fewer meals prepared at home, and increasing consumption of fast food, tobacco and alcohol. The marketing and promotion of unhealthy foods are changing the diets of children. In addition, greater use of motorized transport and the lack of space for walking and biking have reduced physical activity for many people and worsened air quality. All of these factors point towards a dramatic increase in an already high NCD burden.

The Western Pacific Regional Action Plan for the Prevention and Control of Noncommunicable Diseases (2014-2020) was developed in response to a resolution adopted at the sixty-second session of the World Health Organization (WHO), Regional Committee for the Western Pacific. The regional plan is fully harmonized with the Global Action Plan for the Prevention and Control of Noncommunicable Diseases (2013-2020) while adding the value of actions that build on regional achievements, contexts, opportunities and perspective.

## RESPONDING TO THE CHALLENGE OF NONCOMMUNICABLE DISEASES

Effective governance is needed to address social, political and economic pathways that lead to reduction of NCD risk factors and chronic NCD conditions. But political leaders will need information, data and evidence that show how policies impact health and how cost-effective interventions can deliver a higher and more valuable yield in terms of public health vis-à-vis short-sighted economic gains.

It is recognized that countries vary in their infrastructure and in their capacity to implement all policy options and interventions. A set of very cost-effective interventions is summarized in Table 1.

The need for action to combat NCDs has been well articulated. Cost-effective interventions are available. A call for political action at the global level has triggered unprecedented awareness of the urgency of the NCD epidemic. Strategic and specific action that will result in effective policies, programmes and supportive environments is now of critical importance.

## DEVELOPMENT OF THE REGIONAL ACTION PLAN

The Regional Committee for the Western Pacific, in resolution WPR/RC62.R2, requested the Regional Director to develop a regional action plan to address NCDs, in consultation with Member States and in collaboration with partners and stakeholders. The Western Pacific Regional Action Plan for the Prevention and Control of Noncommunicable Diseases (2014-2020) fulfils that mandate and is intended to guide the Region’s governments in strengthening their response to the NCD epidemic. The regional action plan draws upon global commitments contained in the Political Declaration of the High-level Meeting of the General Assembly on the Prevention and Control of Noncommunicable Diseases. The political declaration urges countries to integrate NCD prevention and control into their national health planning process and their development agenda by promoting, establishing or strengthening multisectoral national policies and plans for the prevention and control of NCDs.

## COMPREHENSIVE GLOBAL MONITORING FRAMEWORK FOR THE PREVENTION AND CONTROL OF NONCOMMUNICABLE DISEASES

The comprehensive global monitoring framework, including 25 indicators and a set of nine voluntary global targets for the prevention and control of NCDs, was adopted by the World Health Assembly (WHA66.10) in May 2013 and is presented in Table 2. Countries can develop their national targets in alignment with the global targets. Reports on progress achieved in attaining the nine voluntary global targets will be made in 2016, 2021 and 2026.

## WESTERN PACIFIC REGIONAL ACTION PLAN FOR THE PREVENTION AND CONTROL OF NONCOMMUNICABLE DISEASES (2014-2020)

### Overview

VISION: Governments and societies sustain their political and financial commitments to prevent and control NCDs so that these diseases are no longer a barrier to socioeconomic development.

MISSION: To scale up effective interventions to prevent and control NCDs through health-promoting environments.

GOAL: To reduce the burden of preventable morbidity and disability and avoidable mortality due to NCDs in the Western Pacific Region.

### Overarching principles and approaches

#### Leadership and coordination

Prevention and control of NCDs need a “whole-of-government” and a “whole-of-society” approach. The health sector has to take the lead in evidence-based advocacy and monitoring. Beyond inclusion in national health plans, NCD prevention and control should be included in national development plans.

#### Human rights

NCD prevention and control strategies must be formulated and implemented in accordance with international human rights conventions and agreements.

#### Empowerment of people

Individuals, families, communities and societies should be empowered and involved in activities for the prevention and care of NCDs.

#### Evidence-based practice

Strategies for the prevention and control of NCDs need to be based on scientific evidence and public health principles.

#### Life-course approach

A life-course approach is key to the prevention and control of NCDs. The process starts with maternal health, including preconception, antenatal and postnatal care and maternal nutrition. In addition, proper infant feeding practices, including promotion of breastfeeding and health promotion of children, adolescents and youth, followed by promotion of a healthy working life, healthy ageing and care of NCDs for people in later life, are integral components of a life-course approach.

#### Multisectoral action

Effective NCD interventions require a number of combined elements including, as appropriate, meaningful community participation and engagement, supportive policy prioritization and settings, multisectoral collaboration, a health-in-all-policies approach and active partnerships among national authorities, nongovernmental organizations, academia and private sector.

#### Universal health coverage and equity

Good health is essential to sustained economic and social development and poverty reduction.

Access to needed health services is crucial for maintaining and improving health. At the same time, people need protection from being pushed into poverty because of the cost of health care.

Universal health coverage is defined as ensuring that all people have access to needed promotive, preventive, curative and rehabilitative health services of sufficient quality to be effective, while also ensuring that people do not suffer financial hardship when paying for these services. Universal health coverage has therefore become a major goal for health reform in many countries and a priority objective of WHO.

All people should have full access to health care and opportunities for the prevention and control of NCDs based on need regardless of age, sex, social status, presence of disabilities and the ability to pay.

### Objectives and actions for Member States and WHO

The objectives of the Western Pacific Regional Action Plan for the Prevention and Control of Noncommunicable Diseases (2014-2020) are aligned with Global Action Plan for the Prevention and Control of Noncommunicable Diseases (2013-2020) for consistency and to help Member States adapt them to their national context.

In summary, the objectives are as follows:

1. To raise the priority accorded to the prevention and control of noncommunicable diseases in global, regional and national agendas and internationally agreed development goals, through strengthened international cooperation and advocacy.
2. To strengthen national capacity, leadership, governance, multisectoral action and partnerships to accelerate country response for the prevention and control of noncom-municable diseases.
3. To reduce modifiable risk factors for noncommunicable diseases and underlying social determinants through creation of health-promoting environments.
4. To strengthen and orient health systems to address the prevention and control of noncommunicable diseases and the underlying social determinants through people-centred primary health care and universal health coverage.
5. To promote and support national capacity for high-quality research and development for the prevention and control of noncommunicable diseases.
6. To monitor the trends and determinants of noncommunicable diseases and evaluate progress in their prevention and control.

### Proposed actions for international partners

1. International cooperation and capacity strengthening:
2. Resource mobilization for the prevention and control of NCDs:

### Monitoring and reporting progress

Monitoring and reporting of the Western Pacific Regional Action Plan for the Prevention and Control of Noncommunicable Diseases (2014-2020) will be fully aligned with the proposed monitoring of the Global Action Plan for the Prevention and Control of Noncommunicable Diseases (2013-2020) to harmonize the efforts. WHO is in the process of developing appropriate action plan indicators to monitor progress of implementation of the Global Action Plan for the Prevention and Control of Noncommunicable Diseases (2013-2020). These indicators, based on feasibility, current availability of data and capability of application across the six objectives of the global action plan, will be used to assess the progress made in 2016, 2018, and 2021.

Reports on progress achieved in attaining the nine global voluntary targets will be submitted in 2016, 2021, and 2026.

WHO will also update Annex 3 (menu of policy options) of the global action plan, which appears as Annex 1 of the regional action plan, as appropriate, to be considered through the Executive Board, by the World Health Assembly, in the light of new scientific evidence.

## SYNERGIES BETWEEN NONCOMMUNICABLE DISEASES AND OTHER PROGRAMMES

There are many other conditions of public health importance that are associated with the four main NCDs — cardiovascular diseases, cancers, diabetes and chronic respiratory diseases. The other conditions include:

1. Other NCDs — renal, endocrinal, neurological, haematological, hepatic, gastroenterological, musculoskeletal, skin and oral diseases;
2. Mental disorders;
3. Disabilities, including blindness and deafness;
4. Violence and injuries

Some of these conditions are the subject of other WHO strategies and World Health Assembly resolutions. NCDs and their risk factors are also linked to communicable diseases, maternal and child health, reproductive health, ageing, and social, environmental and occupational determinants of health. The Western Pacific Regional Action Plan for the Prevention and Control of Noncommunicable Diseases (2014-2020) will explore potential synergies between NCDs and interrelated conditions to maximize opportunities and efficiencies for mutual benefit.

## Figures and Tables

**Table 1.** Very cost-effective interventions for the prevention and control of NCDs^1^

Risk factor/disease	Policy options/interventions
Tobacco use	Reduce affordability of tobacco products by increasing tobacco excise taxes
	Create by law completely smoke-free environments in all indoor workplaces, public places and public transport
	Warn people of the dangers of tobacco and tobacco smoke through effective health warnings and mass media campaigns
	Ban all forms of tobacco advertising, promotion and sponsorship
Harmful use of alcohol	Regulate commercial and public availability of alcohol
	Restrict or ban alcohol advertising and promotions
	Use pricing policies, such as excise taxes, on alcoholic beverages
Unhealthy diet	Reduce salt intake, and adjust the iodine content of iodized salt, as appropriate
	Replace trans fats with unsaturated fats
	Implement public awareness programmes on diet
Physical inactivity	Implement public awareness activities to promote the benefits of a physically active lifestyle
Cardiovascular diseases and diabetes	Drug therapy, including glycaemic control for diabetes mellitus and control of hypertension using a total risk approach, for individuals who have had a heart attack or stroke and for people with high risk (30% or higher) of a fatal and nonfatal cardiovascular event in the next 10 years
	Acetylsalicylic acid for acute myocardial infarction
Cancer	Prevention of liver cancer through hepatitis B immunization
	Prevention of cervical cancer through screening, visual inspection with acetic acid (VIA) or Pap smear (cervical cytology) if cost-effective, linked with timely treatment of pre-cancerous lesions

1 Global Action Plan for the Prevention and Control of Noncommunicable Diseases (2013-2020), Appendix 3. Geneva: World Health Organization; 2013.

**Table 2.** Comprehensive global monitoring framework, including 25 indicators, and a set of nine voluntary global targets for the prevention and control of NCDs

Framework element	Target	Indicator
**Mortality and morbidity**		
Premature mortality from noncommunicable disease	(1) A 25% relative reduction in the overall mortality from cardiovascular diseases, cancer, diabetes, or chronic respiratory diseases	(1) Unconditional probability of dying between ages of 30 and 70 from cardiovascular diseases, cancer, diabetes or chronic respiratory diseases
		*Additional indicator*
		(2) Cancer incidence, by type of cancer, per 100 000 population
**Behavioural risk factors**		
Harmful use of alcohol^1^	(2) At least 10% relative reduction in the harmful use of alcohol,^2^ as appropriate, within the national context	(3) Total (recorded and unrecorded) alcohol per capita (aged 15+ years old) consumption within a calendar year in litres of pure alcohol, as appropriate, within the national context
		(4) Age-standardized prevalence of heavy episodic drinking among adolescents and adults, as appropriate, within the national context
		(5) Alcohol-related morbidity and mortality among adolescents and adults, as appropriate, within the national context
Physical inactivity	(3) A 10% relative reduction in prevalence of insufficient physical activity	(6) Prevalence of insufficiently physically active adolescents, defined as less than 60 minutes of moderate to vigorous intensity activity daily
		(7) Age-standardized prevalence of insufficiently physically active persons aged 18+ years (defined as less than 150 minutes of moderate-intensity activity per week, or equivalent)
Salt/sodium intake	(4) A 30% relative reduction in mean population intake of salt/sodium^3^	(8) Age-standardized mean population intake of salt (sodium chloride) per day in grams in persons aged 18+ years
Tobacco use	(5) A 30% relative reduction in prevalence of current tobacco use in persons aged 15+ years	(9) Prevalence of current tobacco use among adolescents
		(10) Age-standardized prevalence of current tobacco use in persons aged 18+ years
**Biological risk factors**		
Raised blood pressure	(6) A 25% relative reduction in the prevalence of raised blood pressure or contain the prevalence of raised blood pressure according to national circumstances	(11) Age-standardized prevalence of raised blood pressure among persons aged 18+ years (defined as systolic blood pressure ≥ 140 mmHg and/or diastolic blood pressure ≥ 90 mmHg) and mean systolic blood pressure
Diabetes and obesity^4^	(7) Halt the rise in diabetes and obesity	(12) Age-standardized prevalence of raised blood glucose/diabetes among persons aged 18+ years (defined as fasting plasma glucose concentration ≥ 7.0 mmol/L (126 mg/dL) or on medication for raised blood glucose)
		(13) Prevalence of overweight and obesity in adolescents (defined according to the WHO growth reference for school-aged children and adolescents, overweight - one standard deviation body mass index for age and sex, and obese - two standard deviations body mass index for age and sex)
		(14) Age-standardized prevalence of overweight and obesity in persons aged 18+ years (defined as body mass index ≥ 25 kg/m² for overweight and body mass index ≥ 30 kg/m² for obesity)
		*Additional indicators*
		(15) Age-standardized mean proportion of total energy intake from saturated fatty acids in persons aged 18+ years^5^
		(16) Age-standardized prevalence of persons (aged 18+ years) consuming less than five total servings (400 g) of fruit and vegetables per day
		(17) Age-standardized prevalence of raised total cholesterol among persons aged 18+ years (defined as total cholesterol ≥ 5.0 mmol/L or 190 mg/dL); and mean total cholesterol concentration
**National systems response**		
Drug therapy to prevent heart attacks and strokes	(8) At least 50% of eligible people receive drug therapy and counselling (including glycaemic control) to prevent heart attacks and strokes	(18) Proportion of eligible persons (defined as aged 40 years and older with a 10-year cardiovascular risk ≥ 30%, including those with existing cardiovascular disease) receiving drug therapy and counselling (including glycaemic control) to prevent heart attacks and strokes
Essential noncommunicable disease medicines and basic technologies to treat major noncommunicable diseases	(9) An 80% availability of the affordable basic technologies and essential medicines, including generics, required to treat major noncommunicable diseases in both public and private facilities	(19) Availability and affordability of quality, safe and efficacious essential noncommunicable disease medicines, including generics, and basic technologies in both public and private facilities
		*Additional indicators*
		(20) Access to palliative care assessed by morphine-equivalent consumption of strong opioid analgesics (excluding methadone) per death from cancer
		(21) Adoption of national policies that limit saturated fatty acids and virtually eliminate partially hydrogenated vegetable oils in the food supply, as appropriate, within the national context and national programmes
		(22) Availability, as appropriate, if cost-effective and affordable, of vaccines against human papillomavirus, according to national programmes and policies
		(23) Policies to reduce the impact on children of marketing of foods and non-alcoholic beverages high in saturated fats, trans fatty acids, free sugars, or salt
		(24) Vaccination coverage against hepatitis B virus monitored by number of third doses of Hep-B vaccine (HepB3) administered to infants
		(25) Proportion of women between the ages of 30-49 screened for cervical cancer at least once, or more often, and for lower or higher age groups according to national programmes or policies

1 Countries will select indicator(s) of harmful use as appropriate to national context and in line with WHO's global strategy to reduce the harmful use of alcohol and that may include prevalence of heavy episodic drinking, total alcohol per capita consumption, and alcohol-related morbidity and mortality, among others.

2 In WHO's global strategy to reduce the harmful use of alcohol the concept of the harmful use of alcohol encompasses the drinking that causes detrimental health and social consequences for the drinker, the people around the drinker and society at large, as well as the patterns of drinking that are associated with increased risk of adverse health outcomes.

3 WHO's recommendation is less than 5 grams of salt or 2 grams of sodium per person per day.

4 Countries will select indicator(s) appropriate to national context.

5 Individual fatty acids within the broad classification of saturated fatty acids have unique biological properties and health effects that can have relevance in developing dietary recommendations.

